# Supplementary material for: Atmospheric modelling of grass pollen rupturing mechanisms for thunderstorm asthma prediction
Source: PLoS One. 2021 Apr 14;16(4):e0249488. doi: 10.1371/journal.pone.0249488 (PMC8046208; doi:10.1371/journal.pone.0249488)
Supplement: S1 File — (DOCX) [file pone.0249488.s001.docx]

**Atmospheric modelling of grass pollen rupturing mechanisms for thunderstorm asthma prediction**

Kathryn M. Emmerson^1^, Jeremy D. Silver^2^, Marcus Thatcher^1^, Alan Wain^3^, Penelope J. Jones^4^, Andrew Dowdy^3^, Edward J. Newbigin^5^, Beau W. Picking^5^, Jason Choi^6^, Elizabeth Ebert^3^_,_ Tony Bannister^3^.

**Supplementary information**

S1 Table Hourly whole grass pollen and pollen shell concentrations measured on 20 and 21 November 2016 at Melbourne University.

| Date and time  UTC | Whole grass pollen  Grains m^-3^ | Pollen shells  Grains m^-3^ |
| --- | --- | --- |
| 20/11/2016 4:30 | 86 | 29 |
| 20/11/2016 5:30 | 80 | 46 |
| 20/11/2016 6:30 | 83 | 59 |
| 20/11/2016 7:30 | 102 | 62 |
| 20/11/2016 8:30 | 107 | 56 |
| 20/11/2016 9:30 | 150 | 102 |
| 20/11/2016 10:30 | 147 | 134 |
| 20/11/2016 11:30 | 177 | 8 |
| 20/11/2016 12:30 | 121 | 67 |
| 20/11/2016 13:30 | 113 | 118 |
| 20/11/2016 14:30 | 147 | 110 |
| 20/11/2016 15:30 | 123 | 91 |
| 20/11/2016 16:30 | 147 | 67 |
| 20/11/2016 17:30 | 123 | 94 |
| 20/11/2016 18:30 | 121 | 62 |
| 20/11/2016 19:30 | 86 | 75 |
| 20/11/2016 20:30 | 70 | 96 |
| 20/11/2016 21:30 | 91 | 70 |
| 20/11/2016 22:30 | 64 | 56 |
| 20/11/2016 23:30 | 78 | 40 |
| 21/11/2016 0:30 | 121 | 40 |
| 21/11/2016 1:30 | 110 | 51 |
| 21/11/2016 2:30 | 110 | 29 |
| 21/11/2016 3:30 | 96 | 70 |
| 21/11/2016 4:30 | 96 | 43 |
| 21/11/2016 5:30 | 131 | 75 |
| 21/11/2016 6:30 | 94 | 48 |
| 21/11/2016 7:30 | 115 | 78 |
| 21/11/2016 8:30 | 40 | 43 |
| 21/11/2016 9:30 | 48 | 35 |
| 21/11/2016 10:30 | 35 | 24 |
| 21/11/2016 11:30 | 46 | 54 |
| 21/11/2016 12:30 | 38 | 21 |
| 21/11/2016 13:30 | 21 | 29 |

S2 Table 24-hourly whole grass pollen measured between 18 - 24 November 2016 at Melbourne University.

| Date and time  UTC | Whole grass pollen  Grains m^-3^ |
| --- | --- |
| 18/11/2016 5:00 | 60 |
| 19/11/2016 5:00 | 22 |
| 20/11/2016 5:00 | 29 |
| 21/11/2016 5:00 | 102 |
| 22/11/2016 5:00 | 19 |
| 23/11/2016 5:00 | 3 |
| 24/11/2016 5:00 | 9 |

S3 Table Details of meteorological models used.

|  | ACCESS | CCAM | WRF |
| --- | --- | --- | --- |
| Boundary conditions | Global ACCESS | ERA Interim | NCEP/FNL |
| Topography | Geoscience Australia digital elevation Mapping | Geoscience Australia digital elevation Mapping | Geoscience Australia digital elevation Mapping |
| Sea surface temperatures | Global Australian Multi-Sensor SST Analysis Daily 0.25° [1] | ERA Interim | Real-Time Global NCEP |
| Boundary layer scheme | Lock et al [2] , ​Edwards et al. [3] | Prognostic turbulence kinetic energy and eddy dissipation [4] | Mellor-Yamada-Janjic scheme [5] |
| Microphysics scheme | Wilson and Ballard [6] single-moment bulk microphysics scheme​; rain as a prognostic variable | Prognostic condensate scheme [7], [8] | Morrison double-moment scheme [9] |
| Radiation | Edwards and Slingo [10] ​with incremental time stepping  scheme [11] | Longwave: Schwarzkopf and Ramaswamy [12] Shortwave: Freidenreich and Ramaswamy [13] | Rapid radiative transfer model (RRTM) [14] |
| Land surface scheme | The Met Office Surface Exchange Scheme version 2 (MOSES2; Essery et al. [15] | Kowalczyk et al [16] | Noah [17] |
| Convection | Gregory and Rowntree [18] | Mass-flux closure | Grell 3D ensemble Scheme [19] |
| Aerosol feedbacks | No | Prognostic aerosols with direct and indirect effects [20] | No |
| Cloud feedbacks | yes Wilson et al. [21] | yes | yes |

**Artificially increasing the modelled whole pollen concentrations at altitude.**

Whether the model vertical whole pollen concentrations are correct or not (and we have no measurements of this in Melbourne), there is concern that the models have not produced enough pollen at the cloud base in order to rupture.

We can test whether increasing the concentration of whole pollen grains at altitude in the models will make any difference to the paper’s main conclusion: that use of an ≥80% relative humidity threshold did not explain the November 2016 Melbourne thunderstorm asthma event.

We have forced the number of pollen grains in all models at 800 hPa to be approximately 2.6 times less than the surface concentrations, similar to measurements made by Damialis et al [22]. We calculate the whole pollen concentration (grains m^-3^) using a negative linear relationship with the CTM model level number, h: whole pollen=-13.8h + 263. The model surface level is 1 and 2000 m is level 12.


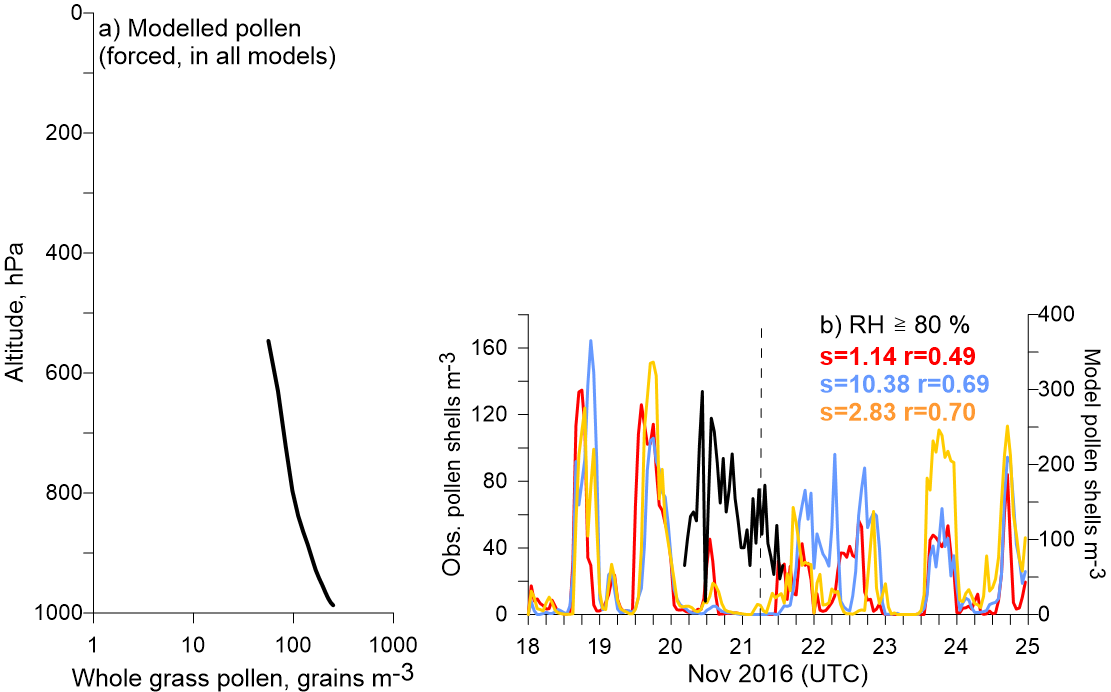


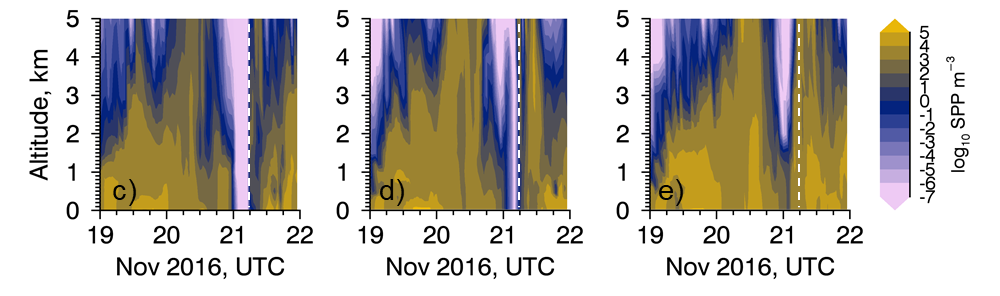


S1 Fig Results of experiment constraining vertical whole pollen concentrations to a surface:2000m ratio of 2.6:1 in all three models (panel a). Panel b shows the time series in pollen shells from the in-atmosphere only RH ≥ 80% experiment. Model values in (b) are on second y-axes to the right. s = slope of the linear regression and r = r correlations between modelled and observed pollen (or pollen shells), given in same colours as the legend. Vertical dashed line indicates the time of the storm. Panels c,d,e show the time series with altitude plots of SPPs to log base 10 in the atmosphere above the pollen counting site in Melbourne. ACCESS^C-CTM^ is shown in S1c, CCAM^C-CTM^ in S1d and WRF^C-CTM^ S1e.

**
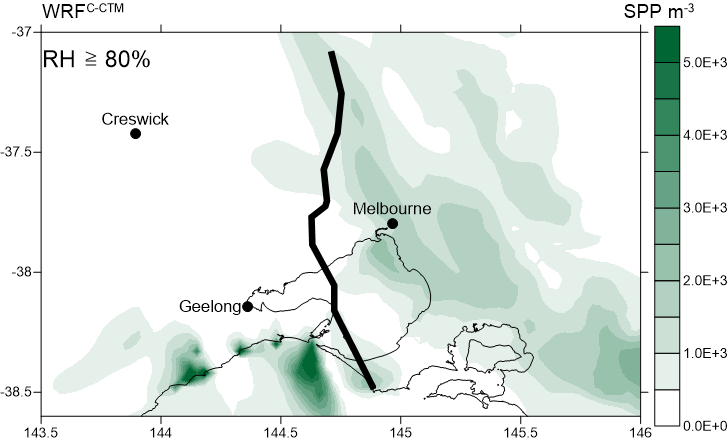
**

S2 Fig Sub-pollen particles (m^-3^) in the surface atmosphere using RH ≥ 80% in WRF^C-CTM^ at 06:00 UTC. Does not include the on-plant mechanical rupturing. The heavy black line shows the approximate position of the storm front diagnosed from radar. Basemap from Igismap.com 2020.


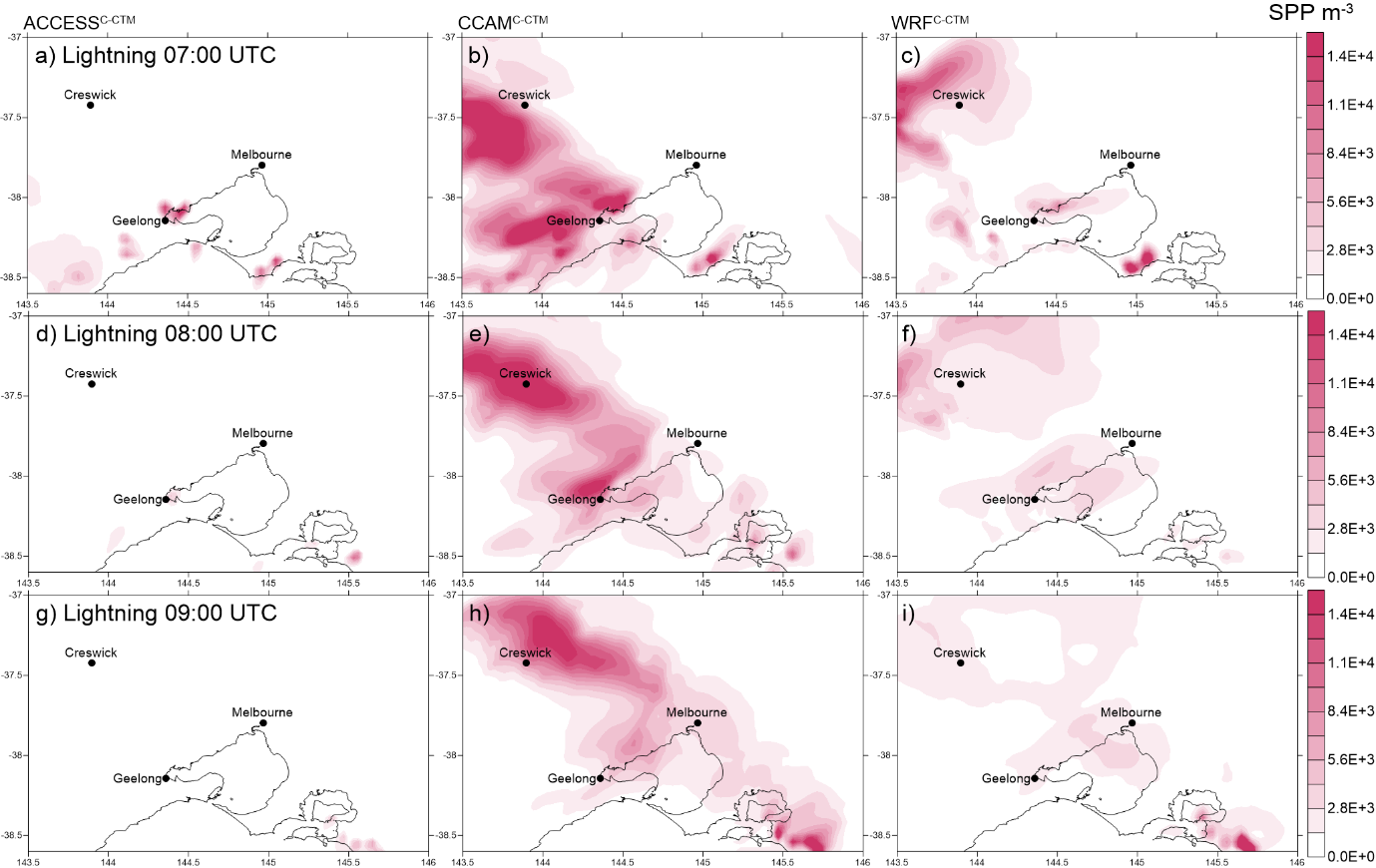


S3 Fig Sub-pollen particles (m^-3^) in the surface atmosphere at 07:00 UTC (panels a,b,c), 08:00 UTC (panels e,f,g) and 09:00 UTC (panels g,h,i) using the lightning mechanism. ACCESS^C-CTM^ is shown in the left hand panels (a,d,g), CCAM^C-CTM^ in the middle panels (b,e,h) and WRF^C-CTM^ in the right hand panels (c,f,i). Basemap from Igismap.com 2020.

**References**

[1] H. Beggs, ‘GAMSSA – A New Global Australian Multi-Sensor SST Analysis, Submitted to Proceedings of the 9th GHRSST–PP Science Team Meeting, 9-13 June 2008.’, Perros-Guirec, France, 2008.

[2] A. P. Lock, A. R. Brown, M. R. Bush, G. M. Martin, and R. N. B. Smith, ‘A New Boundary Layer Mixing Scheme. Part I: Scheme Description and Single-Column Model Tests’, *Mon. Wea. Rev.*, vol. 128, no. 9, pp. 3187–3199, Sep. 2000, doi: 10.1175/1520-0493(2000)128<3187:ANBLMS>2.0.CO;2.

[3] J. M. Edwards, J. R. McGregor, M. R. Bush, and F. J. Bornemann, ‘Assessment of numerical weather forecasts against observations from Cardington: seasonal diurnal cycles of screen-level and surface temperatures and surface fluxes’, *Quarterly Journal of the Royal Meteorological Society*, vol. 137, no. 656, pp. 656–672, 2011, doi: https://doi.org/10.1002/qj.742.

[4] P. Hurley, ‘Modelling Mean and Turbulence Fields in the Dry Convective Boundary Layer with the Eddy-Diffusivity/Mass-Flux Approach’, *Boundary-Layer Meteorol*, vol. 125, no. 3, pp. 525–536, Dec. 2007, doi: 10.1007/s10546-007-9203-8.

[5] Z. I. Janjić, ‘The Step-Mountain Eta Coordinate Model: Further Developments of the Convection, Viscous Sublayer, and Turbulence Closure Schemes’, *Monthly Weather Review*, vol. 122, no. 5, pp. 927–945, May 1994, doi: 10.1175/1520-0493(1994)122<0927:TSMECM>2.0.CO;2.

[6] D. R. Wilson and S. P. Ballard, ‘A microphysically based precipitation scheme for the UK meteorological office unified model’, *Quarterly Journal of the Royal Meteorological Society*, vol. 125, no. 557, pp. 1607–1636, 1999, doi: https://doi.org/10.1002/qj.49712555707.

[7] L. D. Rotstayn, ‘A physically based scheme for the treatment of stratiform clouds and precipitation in large-scale models. I: Description and evaluation of the microphysical processes’, *Quarterly Journal of the Royal Meteorological Society*, vol. 123, no. 541, pp. 1227–1282, 1997, doi: https://doi.org/10.1002/qj.49712354106.

[8] Y.-L. Lin, R. D. Farley, and H. D. Orville, ‘Bulk Parameterization of the Snow Field in a Cloud Model’, *J. Climate Appl. Meteor.*, vol. 22, no. 6, pp. 1065–1092, Jun. 1983, doi: 10.1175/1520-0450(1983)022<1065:BPOTSF>2.0.CO;2.

[9] H. Morrison, G. Thompson, and V. Tatarskii, ‘Impact of Cloud Microphysics on the Development of Trailing Stratiform Precipitation in a Simulated Squall Line: Comparison of One- and Two-Moment Schemes’, *Monthly Weather Review*, vol. 137, no. 3, pp. 991–1007, Mar. 2009, doi: 10.1175/2008MWR2556.1.

[10] J. M. Edwards and A. Slingo, ‘Studies with a flexible new radiation code. I: Choosing a configuration for a large-scale model’, *Quarterly Journal of the Royal Meteorological Society*, vol. 122, no. 531, pp. 689–719, 1996, doi: https://doi.org/10.1002/qj.49712253107.

[11] J. Manners, J.-C. Thelen, J. Petch, P. Hill, and J. M. Edwards, ‘Two fast radiative transfer methods to improve the temporal sampling of clouds in numerical weather prediction and climate models’, *Quarterly Journal of the Royal Meteorological Society*, vol. 135, no. 639, pp. 457–468, 2009, doi: https://doi.org/10.1002/qj.385.

[12] M. D. Schwarzkopf and V. Ramaswamy, ‘Radiative effects of CH4, N2O, halocarbons and the foreign-broadened H2O continuum: A GCM experiment’, *Journal of Geophysical Research: Atmospheres*, vol. 104, no. D8, pp. 9467–9488, 1999, doi: https://doi.org/10.1029/1999JD900003.

[13] S. M. Freidenreich and V. Ramaswamy, ‘A new multiple-band solar radiative parameterization for general circulation models’, *Journal of Geophysical Research: Atmospheres*, vol. 104, no. D24, pp. 31389–31409, 1999, doi: https://doi.org/10.1029/1999JD900456.

[14] E. J. Mlawer, S. J. Taubman, P. D. Brown, M. J. Iacono, and S. A. Clough, ‘Radiative transfer for inhomogeneous atmospheres: RRTM, a validated correlated-k model for the longwave’, *Journal of Geophysical Research: Atmospheres*, vol. 102, no. D14, pp. 16663–16682, 1997, doi: https://doi.org/10.1029/97JD00237.

[15] R. Essery, M. Best, and P. Cox, ‘MOSES 2.2 Technical Documentation’, UK Meterological Office, 2001. Accessed: Nov. 13, 2020. [Online]. Available: https://www.yumpu.com/en/document/view/9128071/moses-22-technical-documentation-pdf-1-mb-met-office.

[16] E. A. Kowalczyk, J. R. Garratt, and P. B. Krummel, ‘Implementation of a soil-canopy scheme into the CSIRO GCM -- regional aspects of the model response’, 1994, doi: https://doi.org/10.4225/08/58655114bd171.

[17] M. Tewari *et al.*, ‘Implementation and verification of the unified NOAH land surface model in the WRF model’, in *In: Paper 14.2A, 20th conference on Weather Analysis and Forecasting /16th conference on numerical weather prediction*, 2004, vol. 1115, p. 6, Accessed: Dec. 07, 2020. [Online]. Available: https://ams.confex.com/ams/pdfpapers/69061.pdf.

[18] D. Gregory and P. R. Rowntree, ‘A Mass Flux Convection Scheme with Representation of Cloud Ensemble Characteristics and Stability-Dependent Closure’, *Mon. Wea. Rev.*, vol. 118, no. 7, pp. 1483–1506, Jul. 1990, doi: 10.1175/1520-0493(1990)118<1483:AMFCSW>2.0.CO;2.

[19] G. A. Grell and D. Dévényi, ‘A generalized approach to parameterizing convection combining ensemble and data assimilation techniques’, *Geophysical Research Letters*, vol. 29, no. 14, pp. 38-1-38–4, 2002, doi: https://doi.org/10.1029/2002GL015311.

[20] L. D. Rotstayn and U. Lohmann, ‘Tropical Rainfall Trends and the Indirect Aerosol Effect’, *J. Climate*, vol. 15, no. 15, pp. 2103–2116, Aug. 2002, doi: 10.1175/1520-0442(2002)015<2103:TRTATI>2.0.CO;2.

[21] D. R. Wilson, A. C. Bushell, A. M. Kerr‐Munslow, J. D. Price, and C. J. Morcrette, ‘PC2: A prognostic cloud fraction and condensation scheme. I: Scheme description’, *Quarterly Journal of the Royal Meteorological Society*, vol. 134, no. 637, pp. 2093–2107, 2008, doi: https://doi.org/10.1002/qj.333.

[22] A. Damialis, E. Kaimakamis, M. Konoglou, I. Akritidis, C. Traidl-Hoffmann, and D. Gioulekas, ‘Estimating the abundance of airborne pollen and fungal spores at variable elevations using an aircraft: how high can they fly?’, *Sci Rep*, vol. 7, p. 44535, 16 2017, doi: 10.1038/srep44535.
