# Supplementary material for: Atmospheric modelling of grass pollen rupturing mechanisms for thunderstorm asthma prediction
Source: PLoS One. 2021 Apr 14;16(4):e0249488. doi: 10.1371/journal.pone.0249488 (PMC8046208; doi:10.1371/journal.pone.0249488)
Supplement: Attachment — Submitted filename: reviewer PONE-D-20-27816.docx [file pone.0249488.s003.docx]

**PONE-D-20-27816**

**Atmospheric modelling of grass pollen rupturing mechanisms for thunderstorm asthma prediction.**

This paper describes an atmospheric modeling approach to investigate the likely mechanisms of pollen grain breakage under the specific conditions of the November 2016 thunderstorm asthma episode in Melbourne. The work is presented in a clear and understandable way. The article is overall pleasant to read and the conclusions are clearly highlighted.

Although the mechanisms of rupture of pollen grains in the aqueous phase or after exposure to moisture are widely documented in the literature, there is still, in my opinion, a lack of validation in real conditions of these mechanisms of allergen release in the fine fraction of the atmospheric aerosol, especially in stormy conditions. The approach by modeling atmospheric conditions and pollen grain rupture conditions is particularly interesting and brings new information for the aerobiology community. This study is the first to compare the amplitude of the different possible mechanisms of pollen grain rupture in real conditions. In my opinion, this work sheds new light on the question of pollen rupture in thunderstorm conditions, which should provoke a discussion in the 'pollen community', and this, even if some choices of parameterization are probably questionable. I am therefore in favor of a publication of this work but I have a series of questions for which I would like to have a discussion with the authors.

I will first outline these important questions that need to be answered and then in a second part I will list some minor points that could improve the article.

NB: I would like to make it clear that my review of the article does not include parameters related to meteorological models for which I am not a specialist.

**Major issues.**

- As I understand it, the value of F_rupt_ is based on Taylor's work (2002) and F_rupt_ has been set at 70%. However, it is not clear in the article whether the F_rupt_ value is the same in the equations in lines L155, L178, L211, L221, L243. If the same value of 0.7 was taken for all rupture mechanisms, how can this be justified when it is a percentage breakage of pollen in water after 5 minutes of immersion. F_rupt_ is for example probably different for mechanical or electrical stress.
- L161 – 166. The explanation of the calculation of n_spg_ seems a little confused to me. SPPs include two main types of particles; starch granules and p-particles. The number of 700 to 1000 SPPs corresponds to starch granules (diameter 1.1 µm) (Suphioglu and Abu Chakra). The value of 106 corresponds to p-particles (diameter 0.3-0.4 µm) whose number is more difficult to estimate (Grote 1994, Heslop-Harrison 1982). The diameter value chosen by the authors seems to me too low (200 nm), and if this value is increased to 400 nm diameter, I am afraid that the number of one million p-particles induces a mass of SPPs greater than that of the whole pollen grain. I would therefore like to have the authors' point of view on these considerations and on their choices concerning the size and number of SPPs per pollen grain.
- Fig. 2b and L364-371. By examining the observed shell pollen concentration, it can be seen that it is not maximum at the time of the storm. Is this measurement of shell pollen with a Hirst reliable? Furthermore, if the rupture mechanism does not allow the presence of aerosolized shell pollen, the concentration of the aerosolized shell pollen in the air will not give any information on the concentration of SPPs.
- The mechanism for on-plant mechanical rupturing is not clear to me. How can the pollen rupture on plants because of wind? Can you please better describe this mechanism L218.

**Minor issues.**

- L151. A reference for the pollen mass is missing.
- Table 1. It would be relevant to add a first column with the description of the rupture mechanism (e.g. 'wet rupture' for RH>80%?). Is the line 'Dust' really useful in this table? Is there a misspelling in line “Mrupt WS” for column “Nrupt” ?
- Fig 1. Flowering grass -> flowering grasses?
- Fig. 5. Add ‘RH’ before %
- Fig 5. Is the unit “log_10_ SPP grains m^-3^” means log_10_ SPPs.m^-3^” ?
- The discussion on figure 8 should have a more comprehensive conclusion. This part is to my opinion less clear than the rest of the paper.
