# Supplementary material for: Atmospheric modelling of grass pollen rupturing mechanisms for thunderstorm asthma prediction
Source: PLoS One. 2021 Apr 14;16(4):e0249488. doi: 10.1371/journal.pone.0249488 (PMC8046208; doi:10.1371/journal.pone.0249488)
Supplement: Attachment — Submitted filename: PlosOnereview2021.docx [file pone.0249488.s005.docx]

PlosOneReview2021

Dear Editor, I realize that authors refused to modify the manuscript following my suggestions . In particular they refuse , in my opinion without motivation, to add two references published in the two most important journals of allergology in the world (JACI and Allergy) . In my opinion these references have importance as a link between pathogenetic and clinical aspects of thunderstorm asthma to clarify the mechanism of rupture of pollen grains during a thunderstorm in pollen seasons and this point is important for the readers. This is the first time, after several years acting as reviewer for the most important medical journals in the world, that I see a total rejection of suggestions of a reviewer by authors without a serious motivation. As a consequence, I can't accept this manuscript can be publiished. Of course, as editor, you can accept or not to publish this paper.

Best regards, Gennaro D'Amato

Gennaro D'Amato, MD, Fellow of AAAAI, FERS , FEAACI. Honorary member of EAACI . Professor of Respiratory Medicine, School of Specialization in Respiratory Diseases, University of Naples Federico II . Chairman Committee World Allergy Organization on "Aerobiology, Climate change and Biodiversity ". Division of Respiratory Diseases and Allergy, High Specialty Hospital A.Cardarelli, Napoli. Member G7 International Committee on “Climate Change and Health” . 2018 WAO-AAAAI Foundation Award and Louis Mendelson Lectureship in Orlando-Florida (USA)

This is my previous revision :Reviewer #3:

Asthma related to thunderstorms (TA) is one of the phenomena that represents a threat to human health which needs to be deeply studied for a correct prevention. Exacerbations of asthma appearing during a thunderstorm in pollen seasons are characterized, at the beginning of the storms, by a rapid increase in visits to the general practitioners or in the emergency departments of hospitals due to attacks of asthma in subjects with allergic IgEmediated sensitization to allergenic pollens (prevalently Gramineae and Parietaria) or mold spores of Alternaria alternata. In the first 20 to 30 minutes of a thunderstorm, patients with pollen allergy can inhale a high concentration of allergens released in atmosphere from pollen grains after the rupture of pollens. Patients without asthma symptoms, but who suffer from seasonal rhinitis may have an asthma attack during a thunderstorm in pollen seasons .

During the events of TA there is a strong association with the elevation of atmospheric concentrations of pollen grains, such as grasses or other species of allergenic plants and symptoms of respiratory allergy and asthma attacks. .

A possible explanation for TA involves the role of rainwater promoting the release of inhalable particles deriving from the rupture of pollen grains. The world’s most severe thunderstorm asthma event occurred in Melbourne, Australia on 21 November 2016, coinciding with the peak of the grass pollen season with 10 deaths and about one twousand persons with asthma attacks.

As mentioned before, the aetiological role of thunderstorms in these events is thought to derive from the rupture of pollens in high humidity conditions, releasing large numbers of sub-pollen particles (SPPs) with pauci-micronic size very easily inhaled deep into the lungs. In this manuscript the authors try to explain the pathogenetic background of TA. The humidity hypothesis was implemented into a three dimensional atmospheric model and driven by inputs from three meteorological models, but the mechanism could not explain how the Melbourne event occurred because relative humidity was very low throughout the atmosphere. Tests in this paper showed humidity induced rupturing occurred frequently at other times and would likely lead to recurrent false alarms if used in a predictive capacity. They used the model to investigate a range of other possible pollen rupturing mechanisms which could have produced high concentrations of subpollen particles in the atmosphere during the storm. The mechanisms studied involve mechanical friction from wind gusts, electrical build up and discharge incurred during conditions of low relative humidity, and lightning strikes.

In particular the mechanisms studied involve mechanical friction from wind gusts, the humidity and the electrical role of thunderbolds ( lightning strikes) . The results of this study suggest that these mechanisms likely operated in tandem with one another, but the lightning method was the only mechanism to generate a pattern in SPPs following the path of the storm. If humidity induced rupturing cannot explain the 2016 Melbourne event, then new targeted laboratory studies of alternative pollen rupture mechanisms would be of value to help constrain the parameterisation of the pollen rupturing process.

In my opinin this is an intersting update on pathogenetic mechanisms of TA but I suggest to add considerations on the clinical importance of these events to better understand the pathogenesis of these event to prevent in future the risk of near fatal asthma and of deaths for asthma.

Considering that these events occurred not only in Australia but exacerbations and asthma epidemics related to thunderstorms have been described in several cities, mainly in Europe (Birmingham and London in the United Kingdom and Naples in Italy), I suggest to add in the references also manuscripts published in journals of American Academy AAAAI such as JACI ( D'Amato G, et al Thunderstorm-related asthma attacks .J Allergy Clin Immunol. 2017 Jun;139(6):1786-1787. doi: 10.1016/j.jaci.2017.03.003. Epub 2017 Mar 23.PMID: 28342913 ) and European Academy EAACI ( D'Amato G, et al . Latest news on relationship between thunderstorms and respiratory allergy, severe asthma, and deaths for asthma. Allergy. 2019;74(1):9-11.) I do not suggest to accept the paper considering that authors haven’t modified the manuscript following my suggestions. You asked my engagemend as reviewer, I worked a lot to read the paper and to suggest some variations and integrations that authors haven’t accepted my suggestions, in particular, they havent’ accepted to integrate references with two recent publications publishd in Journals of American Academy of Allergy, Asthma and Clinical Immunology (JACI) and in the journal of European Academy (Allergy). In my opinion, as invited reviewer , the paper can’t be accepted for publication without this integration of citations.

We are confused by this review. Firstly our manuscript has never been submitted or reviewed anywhere else, and the reviewer has mistaken it for another piece of work they have reviewed. As stated in the covering letter, our manuscript has an atmospheric modelling focus, not on the clinical aspects of asthma. We don’t think the manuscript warrants lengthening with such a clinical discussion.

Reviewer #3 does not have any comments on the science of our manuscript, only that it doesn’t include two of his references. If the editor decides this is an impediment to our publishing this manuscript, then we will include them
