# Supplementary material for: Atmospheric modelling of grass pollen rupturing mechanisms for thunderstorm asthma prediction
Source: PLoS One. 2021 Apr 14;16(4):e0249488. doi: 10.1371/journal.pone.0249488 (PMC8046208; doi:10.1371/journal.pone.0249488)
Supplement: Attachment — Submitted filename: Response to reviewers #2.docx [file pone.0249488.s006.docx]

Response to reviewers. February 2021

Comment from the Editor:

We note that Reviewer 3 has requested that you add several citations to your manuscript, and that you responded in the previous revision as to why you did not feel that these references were appropriate or relevant to your study. Reviewer 4 has agreed with your assessment. As such, please note that further consideration of your manuscript does not depend upon your inclusion of these citations.

Please focus your revisions on the comments provided by Reviewer 4, below.

Thanks you for the opportunity to make a second revision to the manuscript. Our responses to the reviewers are in blue text.

*******************

Reviewer #1: The corrected version completely answers my questions and I consider this paper suitable for publication in PLOS ONE.

However, I have a problem with the "shell/exine" correction suggested by a fellow reviewer. The use of shell in order to refer to the empty pollen enveloppe was correct and I strongly disagree with the use of exine to replace shell. The term shell is used in microencapsulation study, see for example :
Diego–Taboada A, Beckett ST, Atkin SL, Mackenzie G (2014) Hollow pollen shells to enhance drug delivery. Pharmaceutics 6: 80–96
Chapter 24 - Pollen and Spore Shells—Nature’s Microcapsules, Microencapsulation in the Food Industry A Practical Implementation Guide 2014, Pages 283-297
The term exine refers to the outer layer of the pollen wall; it is only a part of the pollen enveloppe (Halbritter et al., Illustrated Pollen Terminology, 2018). I would suggest to use pollen shell, pollen enveloppe, empty (emptied ?) pollen, broken pollen grain, fragmented pollen, but not exine which make incomprehensible some sentences.

We’re happy to return to using pollen ‘shell’. We explain the nomenclature we’re using on line 111 “(called pollen ‘shells’ hereafter)”.

Minor comments.
L283 – the pollen mass should be 2.24 x 10-8 g not 2.24 x 10-9 g (22 ng)

Thanks for picking up this mistake. Changed to 22.4 x 10-9 g to be consistent with description at line 161

Reviewer #2: Review Comments to the Author

I feel that the authors have addressed my concerns and the manuscript acceptable for publication.

Thank you

Reviewer #3: Minor revision (but important for acceptance or rebuttal, see my enclosed comment).

I realize that authors refused to modify the manuscript following my suggestions . In particular they refuse , in my opinion without motivation, to add two references published in the two most important journals of allergology in the world (JACI and Allergy) . In my opinion these references have importance as a link between pathogenetic and clinical aspects of thunderstorm asthma to clarify the mechanism of rupture of pollen grains during a thunderstorm in pollen seasons and this point is important for the readers

As per the editor and other reviewer comments on this issue, we only consider the atmospheric processes in this manuscript.

Reviewer #4: This manuscript presents modeling studies of pollen grains and ruptured pollen (called pollen exine) surrounding the Melbourne, Australia thunderstorm asthma epidemic in 2016. The authors examine the extent to which current pollen rupturing mechanisms documented in the literature can parameterize the occurrence of sub-pollen particles. Overall, the authors reports show that the current understanding of pollen rupturing mechanisms (e.g., humidity > 80%, presence of lightning, wind speeds) are lacking and model predictions do not agree well with experimental observations. The authors conclude that a better understanding of pollen rupturing is needed to support modeling efforts and development of an early-warning system.

The authors have addressed most of the reviewer’s concerns. Additional improvements are needed prior to publication, which are outlined below. I concur with the authors that Reviewer 3’s comments appear are not applicable to the current manuscript.

1. The statistics reported are not yet to the high technical standard required by PLOS ONE. Correlations are widely used as a means of model comparison; however, only Pearson’s correlation coefficients (R values) are reported. When comparing measurement and model results, the slope of the relationship is also needed to compare the magnitude of the model prediction to the experimental data.

Thanks for bringing this to our attention. We have used R to provide all the regression coefficients, standard errors and confidence intervals in a supplementary file. The r values improved as we only compare the period of the observed data with the model. Lower r values were obtained previously as the period of the modelled data extended beyond the observed data.

We have updated figures 2 and 6 to include the slope of the modelled to observed relationships and updated the r values.

2. Line 167: Provide justification for the use of 0.7 for the fraction of whole grass pollen grains that rupture; i.e. that this is the fraction of ryegrass pollen grains that rupture in water after 5 minutes and is the only data available in the literature of its kind.

At line 167: where *F_rupt_* is the fraction of whole grass pollen grains that rupture (=0.7), based on the fraction of ryegrass pollen grains that rupture in water after 5 minutes (15). *F_rupt_* is the same for all model simulations in the absence of literature on ryegrass pollen rupturing under different conditions.

3. Clarification is needed in the discussion of observed and modeled pollen exines. Is it assumed in the model that one exine fragment is produced for one pollen grain?

At line 169. “We assume that 1 empty shell is produced per whole pollen grain”

4. In discussing Figure 2, please comment on the ability of the model to capture the magnitude of pollen exines. It appears that there is a large under-prediction in panel (b) and a large over-prediction in panel (c).

At line 313. “Using the ≥80% RH rupturing mechanism in the models under-predicts the pollen shell concentrations by a factor of ~30 for the in-atmosphere only case, whilst inclusion of the mechanical rupturing overpredicts by a factor of ~80. ”

5. Also in discussing Figure 2 in the paragraph beginning at line 297, the authors should discuss the observations of pollen exine on 20 November. It appears that there is experimental evidence of high concentrations of ruptured pollen grains, although no epidemiological evidence of a thunderstorm asthma outbreak. This seems to suggest that pollen exine is not always linked to negative respiratory outcomes, and that it is not a good measure of allergenic sub-pollen particles that trigger thunderstorm asthma.

We had a discussion on the lack of correlation at line 390. We have moved this section up to line 303 to satisfy query 5.

At line 303 “The peak in pollen shell concentrations (134 grains m^-3^) was measured at 10:30 UTC on 20 November, 20 hours before the storm. Thus there is a lack of correlation between…. ”

6. The discussion of the data from Damailis et al. (2017, doi:10.1038/srep44535) should be improved. In Damailis et al. (2017), Poaceae (grass) pollen had concentrations of 76 grains m-3 at 2000m (measured by aircraft) and 196 grains m-3 at the surface. This corresponds to a 2.6 times higher concentration at the surface compared to 2000 m. The numbers cited by the authors (in Damailis et al. Table 1) correspond to a the “contribution to the total concentration”, which represents the relative abundance of Poaceae pollen relative to other pollen types (17.6% at 2000m and 77.7% at ground level) which is not appropriate for model comparison in this context.

On looking at the Damialis paper again, we have removed this reference from the discussion.

However we note that fig 1f from Damialis et al (2017) does show an exponential reduction in pollen concentrations with elevation in urban environment settings (habitat 1):

**From Damialis et al (2017) :Airborne pollen concentrations per cubic metre of air against habitat type (urban environment, semi-urban and natural ecosystem) and elevation (0–2000 m).** Habitat codes: 1: urban environment, 2: semi-urban environment, 3: natural environment. Elevation codes: 1: lower than 300 m, 2: 301–600 m, 3: 601–900 m, 4: 901–1200 m, 5:1201–1500 m, 6: higher than 1500 m.

7. To resolve the error identified above, the authors should provide a comparable model comparison to these experimental data. This may be done by comparing the ratio of the modeled pollen grain concentration at the surface concentration to that at 2000 m. Observations show the surface is 2.6 times more concentrated than at 2000 m. A glance at Figure 4 panel d indicates the models predict this ratio to be in the range of 50 for WRF, 1000 CCAM, and 10,000 for ACCESS, suggesting a large under-estimation of pollen grain concentrations at 2000m and disagreement between the three meteorological models.

We do not believe that the evidence is clear regarding an error in our vertical distribution of whole pollen as reviewer 4 suggests. Damialis et al also show an exponential reduction in whole pollen for urban environments. We also note the Wozniak et al (2018) model (on which our rupturing experiments were based) also shows this exponential reduction in whole pollen for the urban north east US (fig S4a below):

**From Wozniak et al (2018) Figure S4**: Vertical profiles of average pollen concentrations (grains m^-3^) for **a)** the Northeast and **b)** the Mountain subregions in the SPP_LIT ensemble.

The Damialis ratio for Poaceae is 2.57. In the model (assuming 2000 m is ~800 hPa) these ratios for whole pollen are:

WRF = 277/1.5 = 185

CCAM = 223/0.22 = 1014

ACCESS = 177/0.1 = 1770

Wozniak et al (2018) = ~500, though often less than this outside spring, and with the caveat that their whole pollen contains more than just the grass taxa.

We think the differences derived from use of three meteorological models highlights the strength in producing an ensemble run.

At line 385: “Large differences exist between the models in the whole pollen concentrations produced with altitude and highlights how running an ensemble is beneficial.”

At line 388: “Our models produce whole pollen at 2000 m in the range 185 – 1770 times less than the surface, whereas the Wozniak et al (36) model produces >500 times less in the north east region of the USA in spring.”

8. The authors should reconsider the accuracy of their vertical distributions of pollen grains as a likely source of model error. It appears like the model incorrectly predicts the vertical distribution of pollen grains, keeping them concentrated at the surface and away from higher RH at higher altitude where osmotic rupturing would occur. This is a concern raised by reviewer 4 (comment 24 in the response letter) that has not been adequately addressed in the revised manuscript, because the response incorrectly interprets the results of Damailis et al. (2017) as noted in the earlier comment. Relevant to this topic, are the broader results of Damailis et al. (2017) that airborne concentrations of pollen (at 2000m), measured by aircraft, exceeded those at ground level in the case of 12 of 14 observed pollen species, with Poaceae being one of two pollen types having higher concentrations at ground level. Overall, Damailis et al. (2017) conclude that “our data and the statistical analysis have actually proved lower [pollen and fungal spore] concentrations at lower elevations.”

Acknowledging that we misinterpreted the tabulated Damialis result (though not their figure 1f), we would argue that the region of Thessaloniki is very different to Melbourne and would account for differences in vertical concentrations of pollen. In Melbourne the main grass pollen source region to the west and north is very flat. Damialis et al.'s airborne sampling was done around the coastal city of Thesssaloniki, Greece, which is situated in an area of complex topography (e.g., Mount Chortiatis, which is located about 15km from Thessaloniki has a peak of 1.2 km above sea level). Uplift as winds are coming from inland areas (as the hills appear in most directions around the city) may well enhance the concentration of grass pollen in the air at a height of ~2km (the maximum sampling height in Damialis et al.) relative to what may be observed in an area such as Melbourne.

To be included in the supplementary section:

Whether the model vertical whole pollen concentrations are correct or not (and we have no measurements of this in Melbourne), we think the reviewer’s main concern is that the model has not produced enough pollen at the cloud base in order to rupture.

We can test whether increasing the concentration of whole pollen grains at altitude in the models will make any difference to the paper’s main conclusion: that use of an ≥80% relative humidity threshold did not explain the November 2016 Melbourne thunderstorm asthma event.

We have forced the number of pollen grains in all models at 800 hPa to be approximately 2.6 times less than the surface concentrations, similar to measurements made by Damialis et al (2017). We calculate the whole pollen concentration (grains m^-3^) using a negative linear relationship with the CTM model level number, h: whole pollen=-13.8h + 263. The model surface level is 1 and 2000 m is level 12.

At line 375: Vertical concentrations of whole grass pollen decrease exponentially from the surface, with most (62% average across models) situated in the first 40 m”

At line 379. “Whilst our results, and those of Wozniak et al. (2018) show an exponential decline in whole pollen concentrations with altitude, measurements at 2000m altitude in Thessaloniki, Greece show grass pollen 2.6 times less than the surface concentrations (Damilalis et al., 2017). We tested whether artificially increasing the model vertical whole pollen concentration in the RH ≥ 80% experiment would impact the rate of rupturing at the time of the storm. We applied a linear relationship such that the ratio of surface to 2000 m whole pollen concentrations is 2.6:1. The results are largely similar, with the ≥80% RH threshold producing more pollen shells and SPPs in the models outside of the storm period (and mainly at night) using this mechanism (supplementary).”

9. Line 382 – explain why “this does not make the mechanism a good one for the prediction of thunderstorm asthma”, e.g., because it would lead to false positives.

At line 382: “The high risk of false positives does not make the ≥80% relative humidity mechanism a good one for the prediction of thunderstorm asthma”

10. PLOS ONE requires that all data be made available. It appears that only the radiosonde data is available (line 567), but the 24-h and hourly pollen and pollen exine counts are not listed. Please add these observational data to these data archives, or provide an alternate link to a permanent database.

We have included the hourly pollen measurements as a table in the supplementary section.

Minor comments
11. Line 116: italicized word missing - pollen counts on either side of 21 November

‘on’ added

12. Line 382: Cite the “rupturing experiments cited earlier” here, too, to avoid ambiguity.

Used ‘≥80% RH mechanism’ to avoid further ambiguity.

13. Line 430: Please name the specific “air quality observations.” If not described in the methods of this paper, please provide a reference (e.g., to an online database of hourly PM2.5).

At line 428. …across Melbourne air quality stations (at Footscray, Alphington and Geelong South).

At line 430. the PM2.5 observations at the three Melbourne stations suggest SPPs were too small to contribute much mass to PM_2.5_

Added to the data availability section: Hourly PM10 and PM2.5 observations sourced from Environmental Protection Authority Victoria:<https://discover.data.vic.gov.au/dataset/epa-air-watch-all-sites-air-quality-hourly-averages-yearly/historical>

14. Line 460: Revise “when measurements of SPP concentrations are possible” following recent observations of SPP by Hughes et al. (2020, reference 18).

Changed to “…when observations are available.”

15. Clarify – SPP concentrations should not have units of “grains m-3” because they are not intact grains. A number concentration with units of “m-3” is more appropriate.

Agreed. Two incidences have been changed in the text at lines 487 and 491.

16. Line 523: Clarify – “As we did not determine the concentrations of SPPs….”

This suggests that the technique described by Hughes et al in 2020 was available to us in 2016 and we chose not to use it. Would prefer to change to “We do not have measurements of the SPP concentrations in the Melbourne atmosphere at the time of the storm, therefore we cannot ascertain….”
